# Performance evaluation of the AFIAS IGRA-TB (IFN-gamma) test versus QuantiFERON®-TB gold plus (QFT®-Plus) ELISA in the Barrio Obrero hospital network, Asunción—Paraguay, 2025

**DOI:** 10.3389/fpubh.2025.1729859

**Published:** 2026-01-13

**Authors:** Cynthia Céspedes, Jessica Riveros, Antonio Villalba, Sandra Gutierrez, Roxana Ocampo, Adán Godoy, Sarita Aguirre, Angélica Medina, Guillermo Sequera

**Affiliations:** 1 Programa Nacional de Control de la Tuberculosis, Asunción, Paraguay; 2 Hospital General de Barrio Obrero, Asunción, Paraguay; 3 Research Department, Universidad Central del Paraguay, Pedro Juan Caballero, Paraguay; 4 Cátedra de Salud Pública, Facultad de Ciencias Médicas, Universidad Nacional de Asunción, San Lorenzo, Paraguay

**Keywords:** diagnostic techniques and procedures, interferon-gamma release tests, latent, tuberculosis, point-of-care testing, tuberculosis infection

## Abstract

**Background:**

Tuberculosis infection affects an estimated one-quarter of the global population and represents a key target for TB elimination strategies. While interferon-gamma release assays (IGRAs) such as QuantiFERON-TB Gold Plus (QFT-Plus) are WHO-endorsed, they remain logistically complex in low-resource settings. This study evaluates the performance of the AFIAS IGRA-TB, a qualitative fluorescence immunoassay (FIA), compared to QFT-Plus in Paraguay.

**Methods:**

A cross-sectional diagnostic accuracy study was conducted from January to May 2025 among 210 individuals aged 18–59 years within the Barrio Obrero healthcare network in Asunción, Paraguay. Participants were stratified into three TB risk groups: low-risk (no known exposure), high-risk (close contacts and incarcerated individuals), and active TB cases. Blood samples were tested with both AFIAS and QFT-Plus assays. Discordant results were retested after six to eight weeks. Concordance was assessed using Cohen’s Kappa; quantitative correlations and ROC curves were also analysed.

**Results:**

Of 210 participants, 75.2% were male. Overall positivity rates were 38.5% for QFT-Plus and 37.0% for AFIAS, with an agreement of 89.0% (*κ* = 0.767; *p* < 0.0001). Strong concordance was observed in high-risk groups. Among 23 discordant cases, retesting confirmed the initial AFIAS result in 5 cases and QFT-Plus in 3; 7 remained discordant. Spearman correlation showed strong, significant association between quantitative values. ROC analysis yielded an AUC of 0.890 for AFIAS.

**Conclusion:**

AFIAS IGRA-TB demonstrates comparable performance to QFT-Plus with operational advantages, suggesting it is a viable diagnostic alternative for TB infection, particularly in decentralized or resource-limited settings.

## 1 Introduction

Tuberculosis (TB), caused by *Mycobacterium tuberculosis*, remains a leading cause of death from a single infectious agent worldwide (1). Despite diagnostic and therapeutic advances, the World Health Organization (WHO) estimates that nearly one-quarter of the global population harbors TB infection, which constitutes a substantial reservoir for future active TB cases (2). Interrupting this reservoir through effective identification and treatment of TB infection—especially in high-risk groups—is critical for TB control and eventual elimination (3).

TB infection diagnosis has historically relied on detection of host immune responses (4). The tuberculin skin test (TST), used for decades, has significant limitations including cross-reactivity with the BCG vaccine and environmental mycobacteria, as well as the need for a return visit to read the result (5, 6). Interferon-gamma release assays (IGRAs), which measure cell-mediated responses to *M. tuberculosis*-specific antigens (e.g., ESAT-6 and CFP-10), offer improved specificity. ELISA-based IGRAs such as QuantiFERON-TB Gold Plus are widely used and endorsed by the WHO (7). However, their implementation is often restricted in low-resource settings due to laboratory infrastructure requirements and technical complexity (8).

Recently developed diagnostic tests aim to address these challenges. The AFIAS IGRA-TB is a fluorescence immunoassay (FIA) that qualitatively detects interferon-gamma (IFN-*γ*) and may offer a simpler, faster, and potentially more accessible option for TB infection detection in primary care or decentralized settings. Its deployment could improve access to TB infection diagnosis in high-burden, low-resource environments.

Accessible and reliable TB infection diagnostics are essential for timely intervention in resource-constrained countries, where early detection can substantially reduce morbidity and transmission (9). Recent innovations such as the Xpert MTB/RIF system have already transformed molecular TB diagnostics, underscoring the value of point-of-care (POC) solutions (10).

This study aims to assess the diagnostic performance of the AFIAS IGRA-TB test compared to the WHO-endorsed QuantiFERON-TB Gold Plus assay, in a real-world setting within a high-density, urban neighborhood of Asunción, Paraguay.

## 2 Methods

A cross-sectional diagnostic test evaluation study was conducted between January and May 2025 among individuals of both sexes, aged 18 to 59 years, who belonged to the Barrio Obrero healthcare network in Asunción, the capital city of Paraguay. Paraguay is considered a country with a moderate TB burden, reporting annual incidence rates between 40 and 50 cases per 100,000 population over the past five years (1). Within this setting lies the National Penitentiary Center of Tacumbú, where TB incidence exceeds 3,500 cases per 100,000 persons deprived of liberty annually (11).

To account for the heterogeneity in TB infection prevalence based on participant risk profiles, individuals were categorized into three predefined risk groups for TB infection: Low-risk group (Group 1): included individuals with no known history of TB contact. Participants in this group were personnel from the Asunción Municipality and employees from private companies, including cleaning services and a medical supply distributor. High-risk group (Group 2): included individuals with a recent and intense history of contact with a TB case. This group primarily comprised incarcerated individuals and community members identified as close contacts of confirmed TB patients. Active TB group (Group 3): included individuals diagnosed with bacteriologically confirmed active TB between December 2024 and April 2025. All participants in this group were part of the Barrio Obrero hospital network. Exclusion criteria included individuals undergoing TB treatment for more than 14 days, pregnant women, and patients with autoimmune diseases under immunosuppressive therapy or with a diagnosis of cancer. HIV and diabetes status were recorded for all participants.

The blood samples were collected into two 4-mL lithium-heparin tubes and transported to the laboratory within 3 h. One tube was allocated to the AFIAS IGRA-TB assay, using three tubes (NIL, TB antigen, and mitogen), and the other to the QFT-Plus assay, requiring four tubes (NIL, TB1, TB2, and mitogen). All tubes were incubated at 37 °C for 16 h, followed by centrifugation at 2,000–3,000 rpm for 15 min. QFT-Plus samples underwent ELISA processing (approximately 3 h), whereas AFIAS samples were processed directly on the automated platform (approximately 15 min). Both tests were performed according to the standardized protocols provided by the respective manufacturers. In cases where results from the two tests were discordant, both assays were repeated for the same individual 6 to 8 weeks after the initial testing. The quantitative test values of TB1 and TB2 antigens for QFT-Plus, and TB antigen for FIA were also compared. Complete table of values with sample codes is shared in a Supplementary Table S1.

### 2.1 Statistical analysis

In addition to reporting factors such as age, HIV status, and diabetes, along with the proportion of positive results for each test, the primary analysis focused on evaluating the level of agreement between the qualitative results of the QFT-Plus and AFIAS IGRA-TB assays. Cohen’s Kappa coefficient (*α*) was used to assess concordance. The strength of agreement was classified as follows: <0.20 (poor), 0.21–0.40 (fair), 0.41–0.60 (moderate), 0.61–0.80 (substantial), and 0.81–1.00 (almost perfect). Ninety-five percent confidence intervals (95% CI) were calculated for the Kappa estimates. Quantitative antigen responses for both tests were also analyzed. The Spearman correlation coefficient was calculated to assess the relationship between antigen values, and ROC curve analysis was performed for both assays. The data were analyzed using STATA 18.0 statistical software.

### 2.2 Ethical considerations

All participants provided written informed consent before enrolment and sample collection. Results from the reference test (QFT-Plus) were interpreted following national TB guidelines, and appropriate treatment was initiated according to the recommendations of the National TB Program. Data were anonymized for the purpose of test concordance analysis. The study was approved by the Ethics Committee of the Central Public Health Laboratory of the Ministry of Public Health and Social Welfare of Paraguay (International Certification FWA no. FWA00020088), under approval code CEI-LCSP 258-2024.

## 3 Results

A total of 210 participants were enrolled in the study. The study population was composed of 75.2% males (158/210). The median age was 37 years, with an interquartile range (IQR) of 15. Group 2 included 31 individuals deprived of liberty from the National Penitentiary Center and 9 community contacts of confirmed TB cases. Regarding risk factors, 5 participants reported having type 2 diabetes mellitus, and none declared HIV-positive status, although 34 individuals (16.0%) did not report their HIV status (see Table 1).

**Table 1:** Characteristics of the study population, by TB infection risk groups.

	Overall	Group 1	Group 2	Group 3
	Total	Low risk TBI	High risk TBI	TB case
	*N* = 210	*n* = 110	*n* = 50	*n* = 50
Median (IQR)	37 (15)	44 (21)	33 (11)	32 (12)
Sex				
Male	158	74	37	47
Female	52	36	13	3
Diabetes				
No	155	99	21	35
Yes	37	5	29	3
ND	18	6	0	12
HIV				
No	176	89	43	44
Yes	0	0	0	0
ND	34	21	7	6

ND = no data collected.

Among all participants, 38.5% tested positive with the QFT-Plus assay and 37.0% tested positive with the AFIAS assay. These results reflect an overall agreement of 89.0% between both tests, with a Cohen’s Kappa coefficient of 0.767 and a *p*-value <0.0001 (Table 2). When stratified by risk group, QFT-Plus yielded positive results in 16.4, 48.0, and 78.0% of individuals in Groups 1, 2, and 3, respectively. The AFIAS test showed positivity rates of 18.3, 46.0, and 69.4% for Groups 1, 2, and 3, respectively, with greater agreement observed between both assays in Groups 1 and 2.

**Table 2:** Performance of AFIAS and QFT-Plus tests, overall and by risk groups.

	Overall	Group 1	Group 2	Group 3
	Total	Low risk TBI	High risk TBI	TB case
	*N* = 210	*n* = 110	*n* = 50	*n* = 50
QFT-Plus				
Positives	81	18	24	39
Negatives	129	92	26	11
Indeterminate	-	-	-	-
AFIAS				
Positives	77	20	23	34
Negatives	131	89	27	15
Indeterminate	2	1	-	1
Concordance				
Kappa	0.767	0.658	0.804	0.585
*p*-value	<0.0001	<0.0001	<0.00001	<0.0001
Overall Agreement	89.0%	90.8%	90.0%	83.7%
PPA*	84.0%	77.8%	87.5%	84.2%
NPA°	92.9%	93.4%	92.3%	81.8%
AUC (IC95%)	0.887 (0.784-0.986)	0.806 (0.789-0.981)	0.948 (0.882 – 1.014)	0.880 (0.784-0.976)
Spearman correlation	0.810	0.587	0.886	0.859
*p*-value	<0.0001	<0.0001	<0.0001	<0.0001

*=positive percent agreement; ° = negative percent agreement; AUC = area under curve.

A total of 23 individuals presented discordant results between the two assays. In 8 of these cases, repeat testing at 6 to 8 weeks after the initial testing was not possible. Among the remaining 15 individuals, retesting led to concordant results in 8 cases, confirming the initial AFIAS result in 5 cases and the QFT-Plus result in 3 cases. The remaining 7 individuals continued to show discordant results upon retesting (see Table 3).

**Table 3:** Sequence of outcomes for discordant cases at initial testing and after 6 to 8 weeks.

ID code	Group	QFT-Plus	AFIAS	QFT-Plus 2nd*	AFIAS 2nd*	2nd test agreement
625503	1	Negative	Positive	-	-	Loss of follow-up
642815	1	Negative	Positive	Negative	Positive	Discordance
647900	1	Negative	Positive	Positive	Negative	Discordance
647922	1	**Negative** ^**ǂ**^	Positive	Negative	Negative	Agreement
647926	1	Negative	Positive	Positive	Positive	Discordance
647927	1	**Negative** ^**ǂ**^	Indeterminate	Negative	Negative	Agreement
647936	1	Negative	Positive	-	-	Loss of follow-up
625504	1	Positive	**Negative** ^**ǂ**^	Negative	Negative	Agreement
642824	1	Positive	Negative	-	-	Loss of follow-up
647941	1	Positive	**Negative** ^**ǂ**^	Negative	Negative	Agreement
627837	2	Negative	Positive	Negative	Positive	Discordance
627855	2	Negative	**Positive** ^**ǂ**^	Positive	Positive	Agreement
627850	2	Positive	Negative	Positive	Negative	Discordance
647364	2	Positive	Negative	-	-	Loss of follow-up
658648	2	Positive	Negative	-	-	Loss of follow-up
634211	3	Negative	**Positive** ^**ǂ**^	Positive	Positive	Agreement
652994	3	**Negative** ^**ǂ**^	Positive	Negative	Negative	Agreement
623849	3	Positive	Negative	Positive	Negative	Discordance
623846	3	Positive	Negative	Positive	Negative	Discordance
627888	3	Positive	Negative	-	-	Loss of follow-up
638951	3	Positive	Indeterminate	-	-	Loss of follow-up
640871	3	Positive	**Negative** ^**ǂ**^	Negative	Negative	Agreement
647362	3	Positive	Negative	-	-	Loss of follow-up

*Second test after 6 to 8 weeks of first tests. ^ǂ^ Result from the first test round, confirmed in the second.

The gray shading of some rows indicates that, in the second sample, there was agreement between the two tests.

The values in bold indicate the test that correctly predicted its outcome in the first testing round, which was later confirmed during the test agreement conducted in the second round.

A total of 10 specimens showed different responses between QFT-Plus TB1 and TB2: 6 in the active TB cohort, 1 among contacts, and 3 in a low-risk group. Active TB (*n* = 6): five were TB1-negative/TB2-positive and one was TB1-positive/TB2-negative. Contact (*n* = 1): TB1-negative/TB2-positive. Low-risk group (*n* = 3): two were TB1-negative/TB2-positive and one was TB1-positive/TB2-negative. Among these, 6 specimens showed differences between AFIAS IGRA-TB and QFT-Plus. Of these six QFT–AFIAS pairs with differences, four were retested after 6–8 weeks: two specimens from the low-risk group (both TB1-negative/TB2-positive) converted to QFT TB2-negative, thereby aligning with AFIAS. Two specimens from the active TB cohort (both TB1-negative/TB2-positive) included one that remained unchanged in both assays and one that converted to AFIAS-positive, aligning with QFT TB2. The specimen codes for the 10 analyzed samples are as follows: 623846, 623849, 625504, 627888, 634208, 634218, 647880, 647941, 652992, 669165 (see Supplementary Table S1).

**Figure 1:**
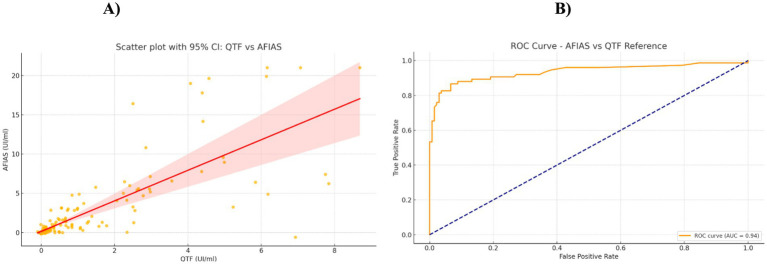
Evaluation of quantitative agreement and diagnostic accuracy between AFIAS and QFT-Plus: **(A)** scatter plot illustrating the correlation of IFN-*γ* concentrations with Spearman’s coefficient = 0.810, *p*-value < 0.0001; **(B)** ROC curve demonstrating test sensitivity and specificity for TB antigen responses with a positivity cutoff of ≥0.35 IU/mL.

Quantitative antigen response values were analysed for both assays. Spearman’s correlation coefficients revealed a strong, positive, and statistically significant correlation between tests, particularly in Groups 2 and 3 (Table 3). ROC curve analysis of AFIAS quantitative values using QFT-Plus as the reference (positive threshold ≥0.35 IU/mL) showed an area under the curve (AUC) of 0.890, indicating high discriminative capacity of the AFIAS assay relative to the QFT-Plus standard (see Figures 1A,B).

## 4 Discussion

This study represents a pioneering and novelty effort to evaluate diagnostic assays for TB infection, using a WHO-endorsed test as a reference standard, in low-resource settings in South America. The primary finding was a high overall agreement of 89.0%, with substantial concordance (*κ* = 0.767), most pronounced in Group 2—individuals recently exposed to TB and at greater risk of progressing to active disease. To our knowledge, no previous study has reported this level of agreement between an AFIAS-based test and QFT-Plus in similarly stratified populations. Risk group stratification is essential, as it allows more accurate interpretation of test performance by contextualizing predictive values.

Our results suggest that the AFIAS IGRA-TB assay is a promising tool for the rapid detection of latent TB infection, especially in low-resource settings that require efficient and affordable diagnostic solutions. Discordant results between the two assays were largely reproduced in the retest conducted 6 to 8 weeks after the initial testing. This reinforces the notion that when AFIAS does not perform optimally, QFT-Plus does not necessarily provide more reliable results either, suggesting that both assays share comparable limitations in borderline or immunologically variable cases (12).

Among the 10 specimens showing discordant QFT-Plus TB1 and TB2 responses clustered near the cutoff, we acknowledge that the limited sample size constrains the analysis. Nevertheless, the observed conversions upon retesting suggest potential biological and/or analytical variability around the diagnostic threshold. These findings highlight the need for cautious interpretation of borderline IGRA results and underscore the importance of considering within-subject variability.

The main limitation of this study is the absence of a definitive gold standard for TB infection diagnosis, which restricts the analysis to concordance rather than true diagnostic accuracy. Additionally, smaller subgroup sizes in Groups 2 and 3 reduced statistical power for some analyses. Nevertheless, the comparison of AFIAS to QFT-Plus—already WHO-endorsed—offers practical validation for real-world implementation. Strengths of the study include a robust sample size of over 200 participants, risk-based stratification, and follow-up testing for discordant cases. While ELISA-based IGRAs like QFT-Plus are known for their high sensitivity and specificity (7), they also present notable operational challenges. The assay requires approximately four hours to complete, often necessitating batch processing to be cost-efficient. It involves multiple manual steps, increasing the risk of human error, and requires specialized readers and software for interpretation—demands that are often prohibitive in decentralized or under-resourced environments. In contrast, the AFIAS platform offers speed and simplicity. This automated system uses single-use cartridges inserted directly into the reader, with minimal user intervention. Full analysis is completed in about 15 min, making it ideal for settings where timely decisions are essential. The device used for this investigation can process up to 6 cartridges simultaneously and delivers printed results without the need for external software or complex interpretation. Besides, the AFIAS line has different platforms with different throughputs that adapts better to the amount of samples tested by a lab. Additionally, due to the cross-sectional design, our analysis focuses exclusively on point-in-time concordance between assays and does not capture clinical outcomes such as progression from latent infection to active TB, this limits our ability to determine which test has superior predictive value for future disease.

In conclusion, our findings support the use of the AFIAS IGRA-TB assay as a reliable and comparable alternative to QFT-Plus for the detection of TB infection. While QFT-Plus pricing varies across countries (approximately USD 20–50 per test), preliminary estimates indicate that AFIAS IGRA-TB may be available at a comparatively lower unit cost in certain settings. Additionally, because AFIAS does not require ELISA automation, specialized maintenance, or consumables associated with microplate processing, it may reduce overall operational expenditures. Its modest infrastructure needs—basic bench space, access to electricity, and room-temperature reagent storage—facilitate deployment in decentralized facilities (7, 12, 13). The platform’s modular design, which supports multiple infectious-disease assays, may also enhance sustainability by distributing equipment costs over time. However, we emphasize that dedicated cost-effectiveness analyses and implementation research are required to fully assess feasibility across diverse health-system contexts.

In other words, the AFIAS IGRA-TB system is more suitable for long-term operation in resource-constrained environments in all aspects, including cost, ease of operation, and supply continuity. Implementing AFIAS testing could facilitate broader TB screening efforts by enabling faster, more accessible identification of individuals with TB exposure—especially those eligible for preventive therapy—thereby contributing to global efforts to end TB (2, 11).

## Data Availability

The raw data supporting the conclusions of this article will be made available by the authors, without undue reservation.
